# Genomic epidemiology of *Streptococcus pneumoniae* reveals globally disseminated lineages and capsular switching in the Arabian Gulf region

**DOI:** 10.3389/fcimb.2026.1832677

**Published:** 2026-09-03

**Authors:** Abiola Senok, Subham Verma, Lobna Mohamed, Eveline Kaambo, Syrine Boucherabine, Madikay Senghore, Anju Nabi, Maya Habous, Fouzia Jabeen, Adnan Alatoom, Najiba Abdulrazzaq, Danesh Moradigaravand, Wafaa Jamal, Rania Nassar, Eiman Mokaddas, Dean B. Everett

**Affiliations:** 1 College of Medicine, Mohammed Bin Rashid University of Medicine and Health Sciences, Dubai, United Arab Emirates; 2 Center for Microbial Sciences, Mohammed Bin Rashid University of Medicine and Health Sciences, Dubai, United Arab Emirates; 3 School of Dentistry, Cardiff University, Cardiff, United Kingdom; 4 Department of Biological Sciences, College of Medicine and Health Sciences, Khalifa University, Abu Dhabi, United Arab Emirates; 5 Department of Public Health and Epidemiology, College of Medicine and Health Sciences, Khalifa University, Abu Dhabi, United Arab Emirates; 6 Biotechnology Center, Khalifa University, Abu Dhabi, United Arab Emirates; 7 Infection Research Unit, College of Medicine and Health Sciences, Khalifa University, Abu Dhabi, United Arab Emirates; 8 Pathology and Laboratory Medicine Department, Dubai Health, Dubai, United Arab Emirates; 9 Purelab Sheikh Khalifah Medical City Laboratories, Sheikh Khalifah Medical City, Abu Dhabi, United Arab Emirates; 10 Al Kuwait Hospital Dubai, Emirates Health Establishment, Dubai, United Arab Emirates; 11 Public Health Sector, Ministry of Health and Prevention, Dubai, United Arab Emirates; 12 Laboratory of Infectious Disease Epidemiology, Biological and Environmental Science and Engineering (BESE) Division, King Abdullah University of Science and Technology (KAUST), Thuwal, Saudi Arabia; 13 Department of Microbiology, College of Medicine, Kuwait University, Kuwait, Kuwait

**Keywords:** antimicrobial resistance (AMR), capsular switching, global pneumococcal sequence clusters, pneumococcal conjugate vaccines, *Streptococcus pneumoniae*

## Abstract

**Background:**

*Streptococcus pneumoniae* remains a major cause of pneumococcal disease globally despite widespread use of pneumococcal conjugate vaccines (PCVs). Genomic data from the Arabian Gulf remain limited, and ongoing changes in pneumococcal population structure may complicate serotype-based surveillance and vaccine impact assessment.

**Methods:**

Whole-genome sequencing was performed on 250 *S. pneumoniae* isolates collected in the United Arab Emirates and Kuwait. Population structure was analysed using Global Pneumococcal Sequence Clusters (GPSCs). Serotypes and sequence types were determined, and capsule switching was assessed by discordance between lineage assignment and capsular type, supported by recombination analysis.

**Results:**

Extensive serotype diversity was observed, comprising 51 capsular serotypes with predominance of Serotype 3 (n=33), followed by serotypes 11A (n=15), 8 (n=12), 23B (n=12), 35B (n=11), and 9N (n=11). The isolates were assigned to 61 Global Pneumococcal Sequence Clusters (GPSCs), with four dominant lineages (GPSC10, GPSC12, GPSC6, and GPSC3) accounting for 28.8% of isolates. GPSC10 (n=27) was the predominant lineage and was mainly associated with serogroup 15 (15B/15C), with all isolates exhibiting multidrug resistance. PCV13 serotypes accounted for 29.2% of isolates, increasing to 50.0% and 65.2% for PCV20 and PCV21, respectively. Five sequence types were associated with two distinct serotypes, consistent with putative capsule-switching events and recombination evidence was identified in one lineage.

**Conclusion:**

Pneumococcal populations in the Arabian Gulf are dominated by globally distributed lineages with substantial serotype diversity. Evidence of capsular switching underscores the limitations of serotype-based surveillance, supporting integration of lineage-resolved genomic surveillance to inform PCV strategies in highly connected regions.

## Introduction

*Streptococcus pneumoniae* remains a leading cause of pneumonia, meningitis, and sepsis globally, especially among children under five and older adults (O'brien et al., 2009; Senok et al., 2023; Mokaddas et al., 2025). The introduction of pneumococcal conjugate vaccines (PCVs) has substantially reduced invasive pneumococcal disease, yet this success has been accompanied by serotype replacement, with non-vaccine serotypes increasingly contributing to pneumococcal disease (Weinberger et al., 2011; Moore et al., 2015; Balsells et al., 2017). These dynamics underpin the development of higher-valency PCV15 and PCV20 vaccines (Hu et al., 2021). The recent introduction of PCV21, which includes eight pneumococcal serotypes not covered by previous vaccines, further expands serotype coverage (Kobayashi et al., 2024).

Patterns of serotype replacement vary and are influenced by vaccine formulation, timing of implementation, antimicrobial use, and population demographics (Weinberger et al., 2011; Balsells et al., 2017). In the Arabian Gulf region, PCVs have been incorporated into national immunization schedules, but pneumococcal genomic surveillance remains limited, with no large-scale whole-genome sequencing (WGS) datasets available (Mokaddas et al., 2025). Recently published work has highlighted substantial gaps in understanding pneumococcal serotypes, lineages, and antimicrobial resistance (AMR) patterns in the United Arab Emirates (UAE), Kuwait, and neighboring states (Senok et al., 2023; Mokaddas et al., 2025).

WGS has transformed pneumococcal epidemiology by enabling high-resolution serotype prediction, detection of capsular switching, delineation of Global Pneumococcal Sequence Clusters (GPSCs), and comprehensive assessment of AMR determinants (Gladstone et al., 2019; Lo et al., 2019). These provide insights not possible with classical serotyping or phenotypic methods. Given the region’s unique demographic structure, including high mobility and diverse expatriate populations, genomic data are essential for evaluating how PCV-driven selection pressures influence pneumococcal populations. The UAE and Kuwait have advanced health systems, and both countries have incorporated PCVs into national immunization programs, primarily using PCV13 during the study period (Mokaddas et al., 2025). This context makes cross-country genomic comparison particularly informative for identifying shared clones, divergent serotype patterns, and regional AMR trends. We present the first WGS of *S. pneumoniae* isolates from these two countries with characterization of serotypes, GPSC lineages, and AMR determinants. This work addresses a major knowledge gap and provides foundational genomic evidence to inform pneumococcal surveillance, AMR monitoring, and vaccine strategy.

## Methods

### Bacterial isolates and study setting

*S. pneumoniae* isolates obtained from the UAE and Kuwait between December 2022 and January 2025 were included in the study. *S. pneumoniae* isolates recovered as part of routine diagnostic work-up from patients with clinical infection were eligible for inclusion irrespective of age or sex. Carriage (colonization) isolates were excluded. Only one isolate per patient was retained, and duplicate isolates from the same patient or infectious episode were excluded. Invasive pneumococcal isolates were *S. pneum*oniae obtained from normally sterile sites, including blood or cerebrospinal fluid, whereas isolates recovered from respiratory samples or other non-sterile sites were classified as non-invasive (https://ndc.services.cdc.gov/case-definitions/invasive-pneumococcal-disease-2017/) (Senok et al., 2023). Both invasive and non-invasive isolates were included in the study to characterize the circulating pneumococcal population associated with clinical infections. The distinction between these groups was retained in the serotype- and vaccine-coverage analyses. Isolate identification at the diagnostic laboratories was performed using standard microbiological methods with the VITEK^®^ 2 system (bioMérieux, Marcy-l’Étoile, France), according to the manufacturer’s instructions. Isolates were stored at −80 °C until analysis.

### DNA extraction and whole-genome sequencing

Samples were pre-treated with metapolyzyme (Sigma-Aldrich, St Louis, MO, USA) to facilitate enzymatic lysis of the Gram-positive cell wall prior to DNA extraction. Genomic DNA was extracted using the DNeasy PowerSoil Pro Kit (Qiagen, Hilden, Germany) and quantified using a Qubit Flex Fluorometer with the Qubit dsDNA HS Assay Kit (Thermo Fisher Scientific, Waltham, MA, USA) (Weinmaier et al., 2023; Oh et al., 2026). Sequencing libraries were prepared using the Watchmaker DNA Library Prep Kit (Watchmaker Genomics, Boulder, CO, USA) according to the validated COSMOSID bacterial isolate whole-genome sequencing workflow (COSMOSID, Germantown, MD, USA) (Oh et al., 2026). Libraries were indexed using xGen Unique Dual Index primers and Stubby Adapters (Integrated DNA Technologies, Coralville, IA, USA), purified with CleanNGS magnetic beads (CleanNA, Waddinxveen, Netherlands), converted to AVITI-compatible circular format using the Element Adept workflow, and sequenced on the Element AVITI platform (2×150 bp; Element Biosciences, San Diego, CA, USA) according to the manufacturers’ instructions (Oh et al., 2026).

### Bioinformatics analysis

Whole-genome sequencing data were processed using the Global Pneumococcal Sequencing (GPS) pipeline (v1.1.0) implemented in Nextflow (v25.04.7). Reads were quality-filtered with fastp (v0.23.4), assembled with Shovill (v1.1.0), and assessed with QUAST (v5.0.2). Draft assemblies were annotated using Prokka (v1.14.6). All samples were subjected to the GPS pipeline’s integrated quality-control framework prior to downstream analysis. Isolates were retained only if they passed assembly-level (total assembly length 1.6–2.3 Mb; mean sequencing depth ≥20×), mapping-level (≥60% reference genome coverage against *S. pneumoniae* ATCC 700669), and taxonomic (≥60% of reads assigned to *S. pneumoniae*) quality thresholds. Per-isolate assembly metrics generated by QUAST, including contig number, total assembly length, and mean sequencing depth, are provided in **Supplementary Data Sheet 1**.

### In silico serotyping, sequence typing, and lineage assignment

In silico serotyping was performed using SeroBA (v2.0.5). Multilocus sequence typing was conducted using MLST (v2.23.0) with assignments based on the *S. pneumoniae* PubMLST database. Population structure was characterized using PopPUNK (v2.6.3) in query-assignment mode against the pre-fitted GPS reference database (v10). Isolates were assigned to Global Pneumococcal Sequence Clusters (GPSCs) using the curated GPS external-cluster designations, with assignment based on the reference model’s established core and accessory genetic distance boundaries. Isolates that could not be confidently matched to an existing GPSC were reported as unassigned.

### Antimicrobial resistance profiling

Genotypic AMR determinants were identified using ARIBA (version 2.14.6; https://github.com/sanger-pathogens/ariba). β-lactam resistance and minimum inhibitory concentration (MIC) predictions were inferred using the CDC PBP AMR Predictor (https://github.com/BenJamesMetcalf/Spn_Scripts_Reference), with interpretation based on CLSI guidelines (Clinical And Laboratory Standards Institute, 2023). Multidrug resistance (MDR) was defined as resistance to three or more antimicrobial classes (Magiorakos et al., 2012).

### Phylogenetic analysis

A recombination-corrected core-genome phylogeny was constructed from Panaroo (v1.5.2, strict mode) core genes aligned with MAFFT. Gene–country associations (UAE vs Kuwait) were assessed using Scoary (v1.6.16) with multiple-testing correction. Recombination was removed using Gubbins (v3.4.3). Maximum-likelihood inference was performed with IQ-TREE (v2.4.0) using ModelFinder and 1,000 ultrafast bootstraps. Trees were visualized in iTOL (v7) with associated epidemiological and genomic metadata.

### Detection of recombination-mediated capsule switching

Core-genome single nucleotide polymorphisms (SNPs) were identified using Snippy (v4.6.0). Recombination was detected and removed using Gubbins (v3.3.1), and recombination-filtered maximum-likelihood phylogenies were inferred with IQ-TREE (v3.0.1). Capsule switching was defined by recombination tracts overlapping the capsular polysaccharide synthesis (cps) locus and visualized using Phandango (v1.3.1). Candidate events were first flagged at the sequence-type level: any MLST sequence type associated with two or more serotypes was recorded as a putative capsule switch. An event was classified as confirmed only if the differing-serotype isolates clustered together in the recombination-masked core-genome phylogeny and Gubbins identified a homologous recombination tract spanning the entire cps locus (including cpsB and cpsD) on the branch separating them, with minimal divergence across the rest of the core genome. Events meeting only the shared-sequence-type criterion were conservatively retained as putative.

### Statistical analysis

All statistical analyses were performed in Python (v3.9.6) using pandas (v2.3.3) and SciPy (v1.13.1). Categorical variables (serotype distributions, vaccine-serotype coverage, and demographic and clinical characteristics) were compared across clinical source (invasive vs non-invasive), age group, and country using two-sided Fisher’s exact tests on 2×2 contingency tables. For serotype-specific comparisons, odds ratios and 95% confidence intervals were estimated. Where a comparison group contained zero observations, the Haldane–Anscombe correction was applied. Vaccine-type coverage (PCV13, PCV15, PCV20, and PCV21) was calculated by age group and invasive/non-invasive and compared using Fisher’s exact tests, including within-stratum comparisons between PCV20 and PCV21 coverage. Gene-wise associations between country of origin and Panaroo-derived gene presence–absence were assessed using Scoary (v1.6.16), with correction for multiple comparisons by both the Bonferroni and Benjamini–Hochberg procedures. A two-sided P value < 0.05 was considered statistically significant throughout. No multivariate regression analysis was performed. Data visualizations were generated using matplotlib (v3.9.4), seaborn (v0.13.2), and the upsetplot package (v0.9.0). Phylogenetic trees were visualized and annotated in iTOL (v7), and recombination tracts relative to the cps locus were visualized using Phandango (v1.3.1).

## Results

We analyzed 250 *S. pneumoniae* isolates, comprising 153 (61.2%) from the UAE and 97 (38.8%) from Kuwait. The majority of isolates were from adult patients (n/N=155/250; 62%) with a preponderance of males (n/N=159/250; 63.6%). A total of 103 isolates were associated with invasive disease and recovered from sterile sites (blood or cerebrospinal fluid) while the non-invasive isolates were predominantly from sputum, nasal swab and endotracheal aspirate. **Table 1** shows the patient demographic distribution and proportion of invasive isolates across the two countries.

**Table 1:** Patient demographics and clinical profile.

Characteristic	Category	Total (n=250)	UAE (n=153)	Kuwait (n=97)	P-value*
Country	UAE	153 (61.2%)	–	–	–
	Kuwait	97 (38.8%)	–	–	–
Sex	Male	159 (63.6%)	93 (60.8%)	66 (63.9%)	0.34
	Female	89 (35.6%)	58 (37.9%)	31 (35.1%)	
	Unknown	2 (0.8%)	2 (1.3%)	0 (0.0%)	
Age group	Adul (≥18 years)	155 (62.0%)	102 (66.7%)	53 (54.6%)	0.062
	Pediatrics (<18 years)	95 (38.0%)	51 (33.3%)	44 (45.4%)	
Age subgroup	<2 years	43 (17.2%)	13 (8.5%)	30 (30.9%)	–
Clinical infection profile	Invasive	103 (41.2%)	89 (58.2%)	14 (14.4%)	<0.001
	Non-invasive	147 (58.8%)	64 (41.8%)	83 (85.6%)	

*P-values were calculated using Fisher’s exact test for comparisons between UAE and Kuwait. For variables with multiple categories, a single p-value reflects the overall comparison across categories.

### Serotype distribution overall, by country, age group and invasiveness

**Figure 1:**
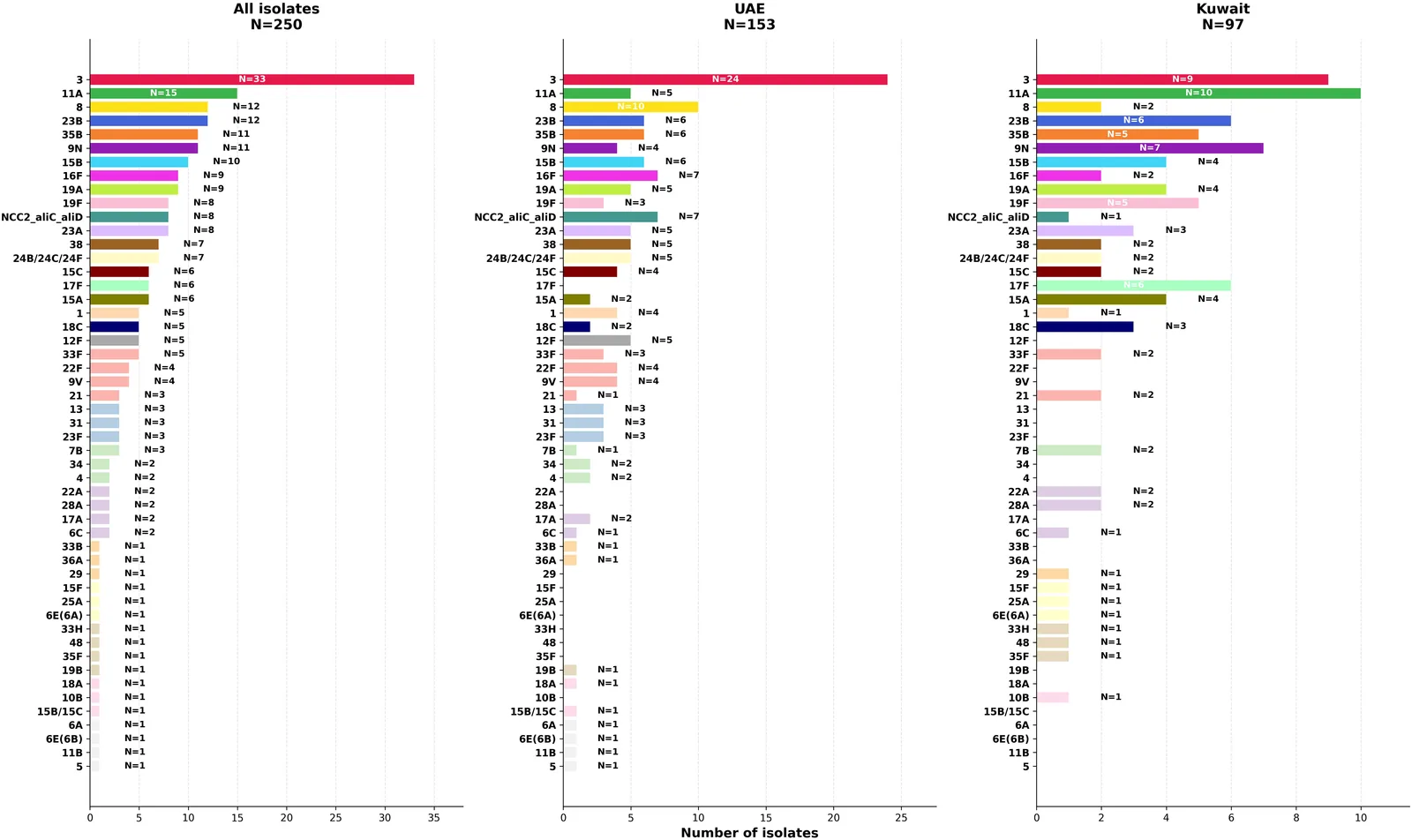
Serotype distribution among *Streptococcus pneumoniae* isolates, overall and by country. Overall and country-specific distribution of serotypes among all isolates (N = 250), including combined dataset (N = 250), United Arab Emirates (UAE; N = 153), and Kuwait (N = 97). Bars represent the number of isolates per serotype.

**Figure 2:**
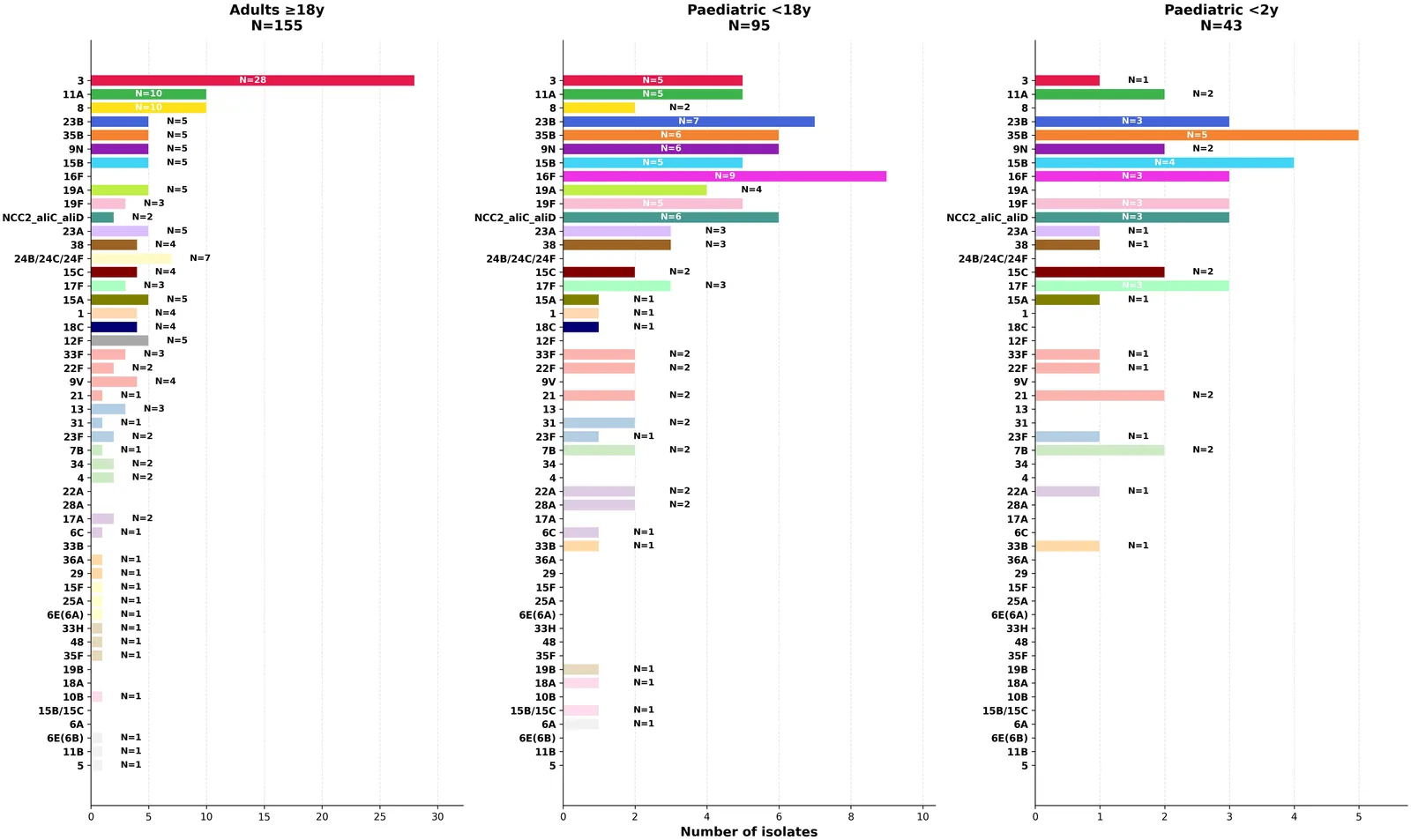
Serotype distribution among *Streptococcus pneumoniae* isolates by age group. Distribution of serotypes stratified by age group: adults (≥18 years; N = 155), paediatric (<18 years; N = 95), and children <2 years (N = 43). Bars represent the number of isolates per serotype within each age category. Only serotypes detected in the respective subgroup are shown.

**Figure 3:**
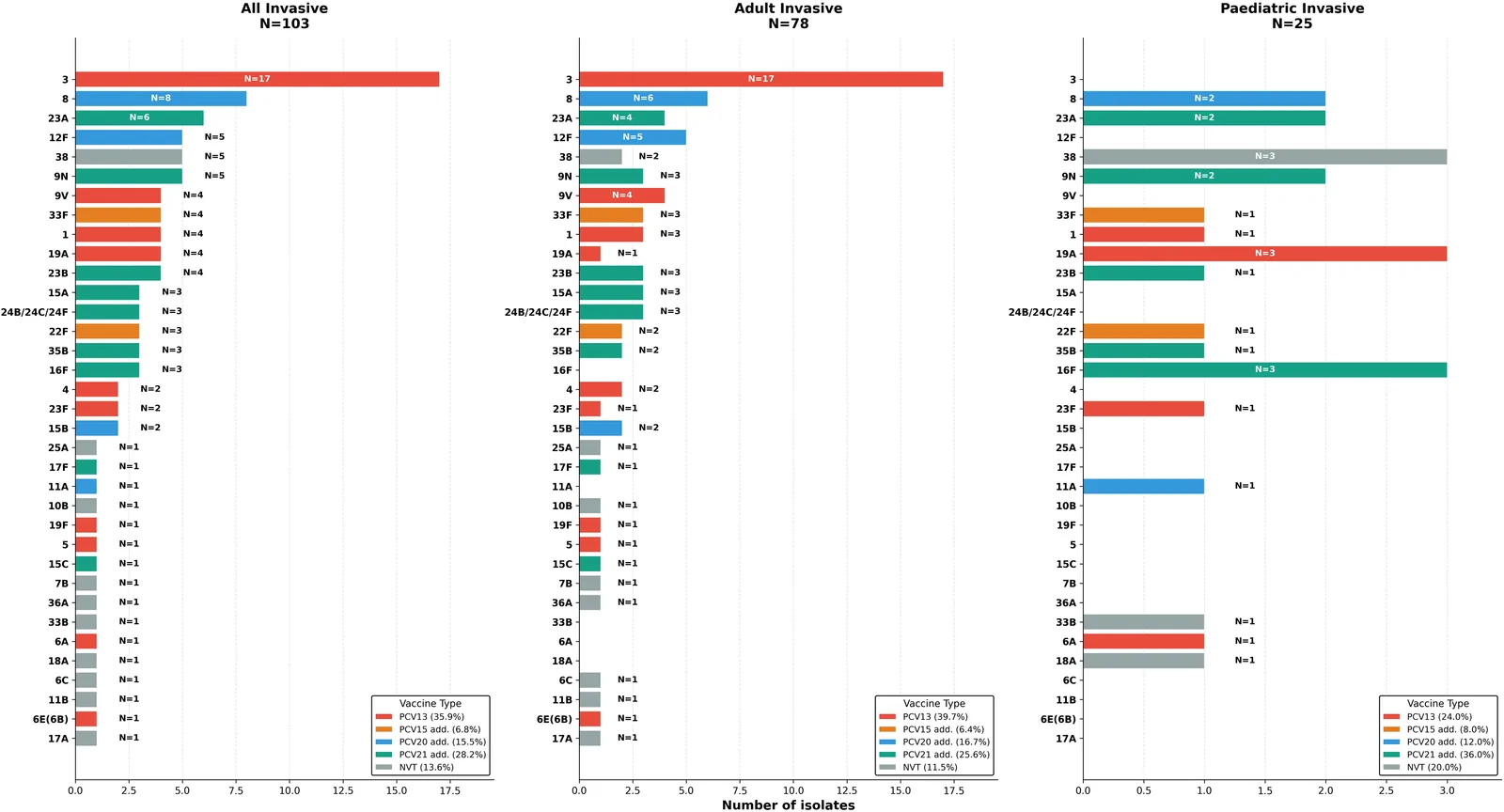
Distribution of invasive *Streptococcus pneumoniae* serotypes by PCV formulation coverage. Horizontal bar charts show the frequency of serotypes causing invasive disease overall (All invasive, N = 103), among adults (Adult invasive, N = 78), and among children (Paediatric invasive, N = 25). Bars are color-coded according to vaccine formulation coverage: PCV13 serotypes, additional serotypes included in PCV15, PCV20, and PCV21, and non-vaccine serotypes (NVT). Percentages shown in the legend represent the proportion of isolates covered by each vaccine category within the respective group.

Across the dataset, 51 distinct capsular serotypes and eight non-encapsulated isolates were identified (**Figure 1**). Overall, serotype 3 was the most prevalent (n=33), followed by serotypes 11A (n=15), 8 (n=12), 23B (n=12), 35B (n=11), and 9N (n=11) (**Figure 1**). Country-specific analysis demonstrated differences in both serotype composition and richness (**Figure 1**). The number of distinct serotypes was higher in the UAE than in Kuwait (40 vs 33). In the UAE, serotype 3 predominated, followed by serotypes 8 and 16F. In Kuwait, serotype 11A was the most common, followed by serotypes 3 and 9N. Serotype distributions also varied substantially by age group (**Figure 2**). Among adults, serotype 3 (n/N=28/155; 18.1%) predominated, followed by serotypes 11A and 8. In contrast, pediatric isolates exhibited a more heterogeneous distribution, with serotypes 16F, 23B and 9N most frequently observed. Among children <2 years of age, serotype diversity was more restricted, with serotypes 35B and 15B accounting for the largest proportions, followed by 19F and 17F. Across invasive pneumococcal isolates, a substantial proportion of circulating serotypes remain covered by PCV15. Overall, 42.7% of all invasive isolates (N = 103) corresponded to PCV15 serotypes, comprising 35.9% PCV13 serotypes and an additional 6.8% uniquely covered by PCV15 (**Figure 3**). Among adults with invasive isolates, PCV15-covered serotypes accounted for 46.1% of isolates (39.7% PCV13 plus 6.4% PCV15-additional), whereas in children, the proportion was lower at 32.0% (24.0% PCV13 plus 8.0% PCV15-additional).

Serotype-specific significant associations with invasiveness and age variables are summarized in **Supplementary Table 1**. Several serotypes showed differential distributions between invasive and non-invasive cases, across age groups, and in early childhood (<2 years). Serotypes 12F and 9V were detected exclusively in invasive disease, occurring in 5 (4.9%) and 4 (3.9%) of the 103 invasive isolates, respectively, while neither serotype was detected among the 147 non-invasive isolates (both p<0.05). In contrast, serotype 11A and non-encapsulated isolates were associated with non-invasive infections. Serotype 11A was identified in 14/147 (9.5%) non-invasive isolates compared with 1/103 (1.0%) invasive isolates (OR for invasive disease 0.09, 95% CI 0.01–0.72; p=0.005), while non-encapsulated isolates were detected only among non-invasive isolates (n/N=8/147; 5.4%) (**Supplementary Table 1**). Age-stratified analysis demonstrated clear serotype heterogeneity. Serotype 16F was significantly more frequently detected among paediatric isolates (n/N=9/95; 9.5% vs n/N=0/155 in adults; p=0.0001). In contrast, serotype 3 (n/N=28/155; 18.1% vs n/N=5/95; 5.3%; OR 3.97, 95% CI 1.48–10.67; p=0.003) and serogroup 24 (n/N=7/155; 4.5% vs n/N=0/95; p=0.046) were significantly more common among adult isolates (**Supplementary Table 1**). Serotype 35B was overrepresented among children <2 years of age (n/N=5/43; 11.6%) compared to other age groups (n/N=6/207; 2.9%]; OR 4.41, 95% CI 1.28–15.18; p=0.02), whereas several serotypes common in adults were infrequent in this youngest age group (**Supplementary Table 1**). Full serotype-level results are presented in **Supplementary Data Sheet 2**.

### Global pneumococcal sequence clusters assignment

**Figure 4:**
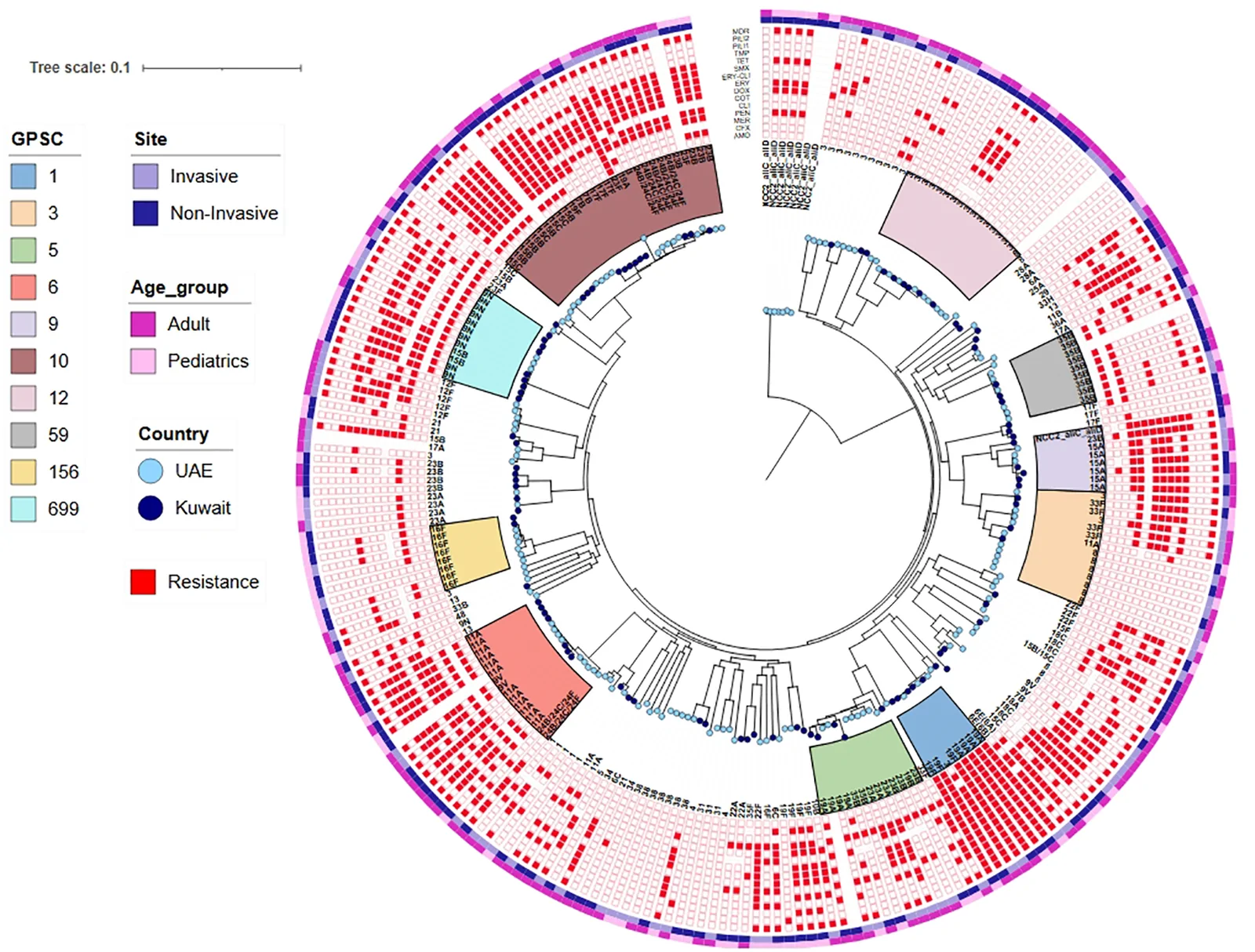
Phylogenetic structure of *Streptococcus pneumoniae* isolates by country, serotype, lineage, and resistance profile. Midpoint-rooted, recombination-masked core genome phylogeny of 250 pneumococcal isolates. Terminal nodes are coloured according to country of origin (United Arab Emirates and Kuwait), and the ten most prevalent Global Pneumococcal Sequence Clusters (GPSCs) are highlighted, with corresponding antimicrobial resistance profiles indicated for each lineage. Notably, all isolates belonging to GPSC1 were uniformly positive for the full complement of detected antimicrobial resistance determinants.

The pneumococcal genomes encompassed 61 GPSCs, with 12 isolates unassigned. Four GPSCs, namely GPSC10, GPSC12, GPSC6, and GPSC3, accounted for 72 of 250 isolates (28.8%) and made a disproportionate contribution to MDR, invasive disease, and the circulation of non-vaccine serotypes (**Table 2**). GPSC10 (n=27) was the predominant lineage and was mainly associated with serogroup 15 (serotypes 15B and 15C). These were detected in both the UAE and Kuwait, and all isolates were MDR. In contrast, GPSC12 (n=16) was strongly associated with serotype 3 and included both invasive and non-invasive isolates. GPSC6 (n=15) was primarily associated with non-PCV13 serotypes, including serotype 11A, and showed a marked MDR profile. GPSC3 (n=14) encompassed multiple serotypes, including 8 and 9N, and was detected across invasive and non-invasive isolates, reflecting underlying genetic heterogeneity. All the major GPSCs included isolates from both countries (**Figure 4**). Although some clusters showed modest country-specific enrichment (for example, GPSC699 in Kuwait), there was no evidence of strict phylogeographic structuring (**Figure 4**). The phylogeny demonstrates extensive lineage diversity with intermixing of isolates from both countries and the concentration of MDR and virulence-associated traits within specific high-risk GPSCs (**Figure 4**).

**Table 2:** Distribution and characteristics of dominant global pneumococcal sequence Clusters (GPSCs) by clinical source, serotype, and vaccine inclusion.

GPSC	Isolates # (%)	Invasive isolates	Non-invasive isolates	Dominant serotype(s)	PCV13	PCV15	PCV20	PCV21
GPSC10	27 (10.8)	14	13	15B, 15C	✗	✗	✓	✓
GPSC12	16 (6.4)	9	7	3	✓	✓	✓	✓
GPSC6	15 (6.0)	6	9	11A	✗	✗	✓	✓
GPSC3	14 (5.6)	5	9	8, 9N	✗	✗	✓	✓

### Pan-genome structure and gene content variation between country isolates

Country-stratified pan-genome analysis showed identical total gene repertoire of 4,316 genes in both the UAE (n=153) and Kuwait (n=97) isolate collections (**Supplementary Table 2**). Using a ≥95% prevalence threshold to define core genes, the number of core genes was higher among Kuwait isolates (1,550 vs 1,418). In contrast, UAE isolates exhibited a larger shell genome (875 vs 702 genes), consistent with greater lineage diversity captured in the larger dataset. The number of cloud genes was comparable between countries (2,023 vs 2,064), suggesting similar levels of rare gene content and no evidence of country-specific enrichment of low-frequency genes. To further assess potential differences in gene profiles between countries, we performed a gene-wise association analysis using Scoary based on Panaroo-derived gene presence–absence matrices, with country of origin (UAE vs Kuwait) as a binary trait. Although multiple genes showed nominal associations with country based on unadjusted p-values, none remained significant after correction for multiple testing using either Bonferroni or Benjamini–Hochberg procedures.

### Serotype distribution relative to pneumococcal conjugate vaccine formulations

**Figure 5:**
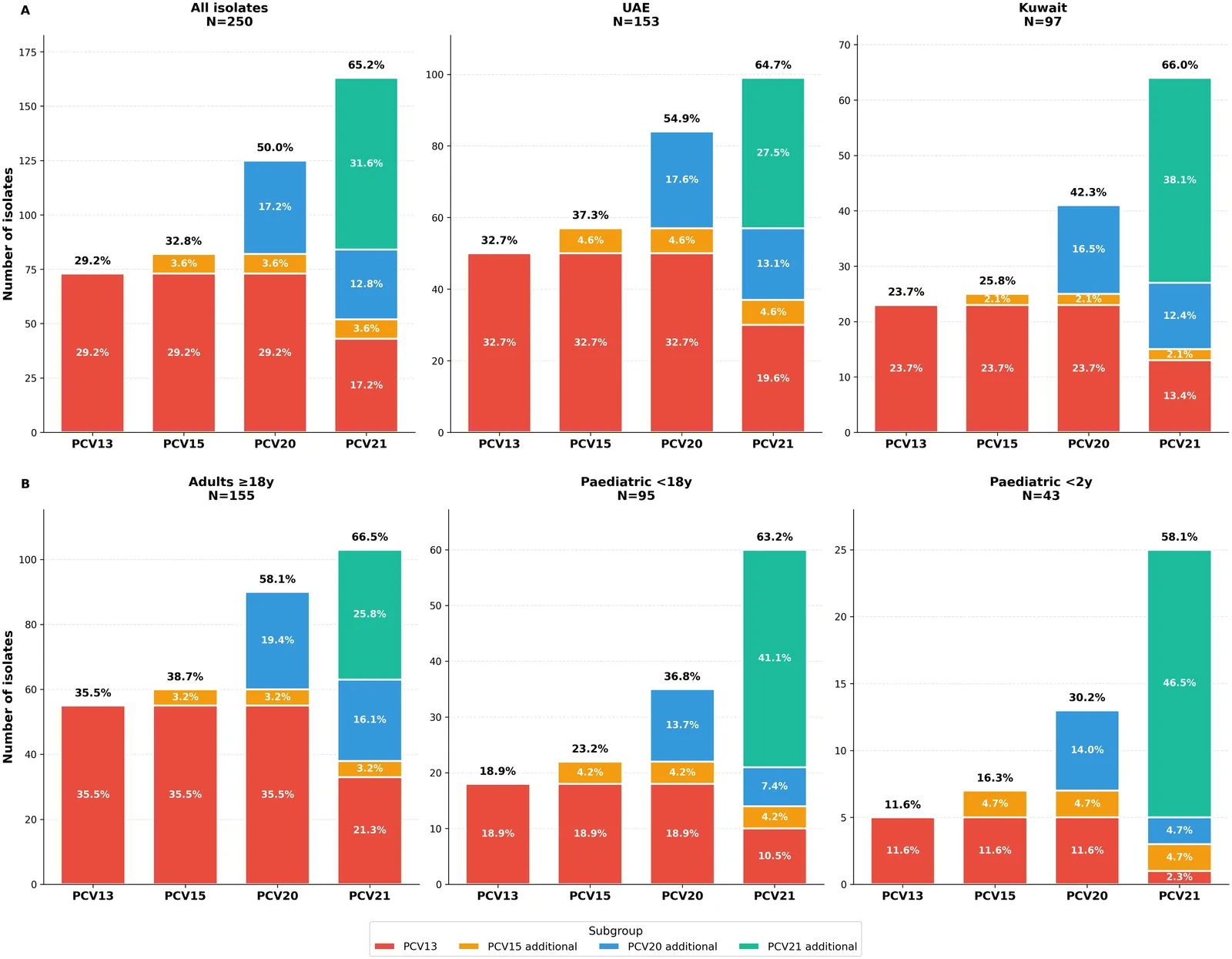
Serotype distribution relative to coverage of pneumococcal conjugate vaccines. **(A)** Stacked bar charts showing the number and proportion of isolates covered by PCV13, PCV15, PCV20, and PCV21 for all isolates, isolates from the UAE and Kuwait respectively. **(B)** Stacked bar charts showing the number and proportion of isolates covered by PCV13, PCV15, PCV20, and PCV21 stratified by age group (adults ≥18 years, children <18 years, and children <2 years). Bars are color-coded to indicate serotypes included in PCV13 and additional serotypes included in PCV15, PCV20, and PCV21. Percentages indicate the proportion of isolates covered by each vaccine formulation within each subgroup.

The distribution of pneumococcal serotypes relative to currently available PCV formulations is shown in **Figures 5A, B** and **Supplementary Table 3**. Overall, PCV13 serotypes accounted for 29.2% (n/N=73/250) of isolates, increasing modestly to 32.8% (n/N=82/250) when PCV15 serotypes were included. Coverage increased substantially with higher-valency formulations, with PCV20 and PCV21 serotypes accounting for 50.0% (n/N=125/250) and 65.2% (n/N=163/250) of isolates, respectively (**Figure 5A**). Country-level analysis demonstrated broadly similar trends in vaccine serotype coverage between the UAE and Kuwait, with incremental gains observed with increasing vaccine valency (**Figure 5A**). Vaccine serotype coverage also varied markedly by age group (**Figure 5B**). Among adults, PCV13 and PCV15 serotypes accounted for 35.5% and 38.7% of isolates, respectively, increasing to 58.1% for PCV20 and 66.5% for PCV21. In contrast, pediatric isolates showed substantially lower coverage for PCV13 (18.9%) and PCV15 (23.2%), with coverage increasing to 36.8% for PCV20 and 63.2% for PCV21. The greatest disparity was observed among children <2 years of age, in whom PCV13 serotypes accounted for 11.6% of isolates, compared with 58.1% for PCV21 (**Figure 5B**).

Stratification by age group and clinical presentation showed differences in serotype coverage between PCV20 and PCV21 (**Supplementary Table 4**). Overall, coverage was higher for PCV21 relative to PCV20 (65.2% vs 50.0%; p<0.001). Among paediatric cases, higher coverage with PCV21 was observed for both invasive (72.0% vs 44.0%; p=0.085) and non-invasive isolates (60.0% vs 34.3%; p=0.004). In children under two years of age, higher coverage with PCV21 was observed for non-invasive isolates (62.5% vs 30.0%; p=0.007), whereas no difference was observed for invasive isolates, likely reflecting the small number of cases. In adults, coverage was higher for PCV21 than PCV20 for both invasive and non-invasive isolates; however, these differences were not statistically significant.

### Capsular switching within sequence types

**Figure 6:**
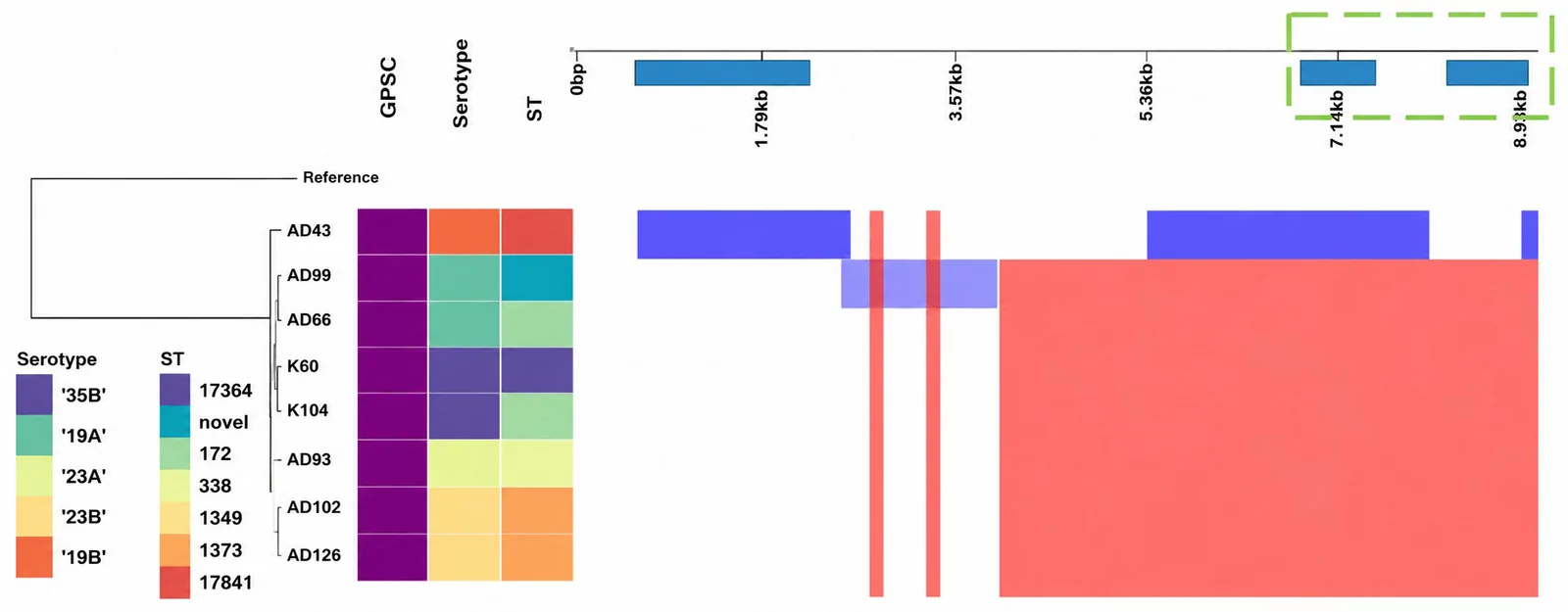
Capsular switching within sequence types. Recombination-masked core genome phylogeny of GPSC5 isolates is shown on the left, with associated metadata tracks indicating GPSC assignment, serotype, and sequence type (ST). Genome-wide homologous recombination events inferred by Gubbins are shown as red blocks across the reference genome coordinates. The capsular polysaccharide synthesis (cps) locus is highlighted in green, and gene annotations (blue blocks) indicate the positions of cps regulatory genes, including cpsB and cpsD. Two closely related ST172 isolates, AD66 (serotype 19A) and K104 (serotype 35B), differ by cps-overlapping recombination events, demonstrating a recombination-mediated capsule switch.

Five sequence types were each identified in association with two distinct serotypes, consistent with putative capsule-switching events (**Supplementary Table 5**). These included ST63 (GPSC9; serotypes 15A and 23B), ST320 (GPSC1; 19A and 19F), ST172 (GPSC5; 19A and 35B), ST6359 (GPSC699; 9N and 15B), and ST13727 (GPSC10; 15B and 15C) (**Supplementary Table 5**). In several instances, the paired serotypes comprised vaccine and non-vaccine types. Among these five events, only GPSC5 showed clear cps locus overlapping recombination tracts, providing robust evidence for recombination-mediated capsule switching (**Figure 6**). The remaining events lacked definitive cps-spanning recombination signals and were therefore classified as putative (**Supplementary Table 5**). Within GPSC5, two closely related isolates, AD66 (serotype 19A) and K104 (serotype 35B), both belonging to ST172, clustered together in the recombination-masked core genome phylogeny, consistent with a shared genetic background. Notably, the serotype 19A isolate was collected in 2022, whereas the serotype 35B isolate was recovered in 2024, providing a temporal framework consistent with a recent capsule-switching event. Despite their close relatedness, the isolates expressed distinct capsular serotypes with homologous recombination tracts spanning the *cps* locus on the branch separating them, whilst the surrounding core genome showed minimal divergence. The entire cps locus, including key regulatory genes such as *cpsB* and *cpsD*, was within the inferred recombination tracts (**Figure 6**).

### Antimicrobial resistance and lineage-level distribution of multidrug resistance

Overall, 154 of 250 isolates (61·6%) exhibited genomic profiles consistent with MDR. Resistance determinants to β-lactams, macrolides, and tetracyclines were most common, whereas carbapenem and fluoroquinolone resistance genes were rare or absent. MDR was unevenly distributed and strongly structured by lineage. Several globally disseminated lineages, particularly GPSC1, GPSC10, GPSC6, GPSC9, and GPSC699, were uniformly MDR (**Figure 4**), while others (GPSC3, GPSC5, GPSC12) comprised both MDR and non-MDR isolates. Combinatorial analysis showed that MDR was driven by a limited number of recurring resistance-class combinations rather than diverse unique profiles (**Supplementary Figure 1**).

## Discussion

This study provides an integrated genomic and epidemiological characterization of contemporary *S. pneumoniae* isolates circulating in two countries in the Arabian Gulf region. While many features of the pneumococcal populations observed in Kuwait and the UAE are consistent with global post-PCV trends, our findings suggest a high degree of epidemiological connectivity among the circulating pneumococcal populations in the two countries. Despite being independent national health systems, isolates from both settings shared identical pan-genome structures, overlapping GPSCs, and absence of robust country-specific genomic signatures. This absence of phylogeographic structuring indicates that population mobility, expatriate-dominant demographics, and shared healthcare-associated selective pressures outweigh national boundaries in shaping pneumococcal population structure.

Serotype diversity was high, with 51 capsular serotypes and non-encapsulated pneumococci identified. Similar patterns of heterogeneity have been reported globally following widespread PCV implementation, where reductions in vaccine-type disease have been accompanied by expansion of non-vaccine serotypes rather than contraction of overall diversity (Croucher et al., 2011; Lo et al., 2019). Serotype 3 was the most prevalent overall, particularly from adult patients. This is in keeping with evidence suggesting a limited impact of vaccination on pneumococcal disease associated with serotype 3 (Silva-Costa et al., 2024; Smaoui et al., 2025; Zintgraff et al., 2026). Several features of serotype 3 may underlie this persistence. Its capsule is synthase-dependent and only loosely cell-associated, with shedding of free polysaccharide that can neutralise anticapsular antibody and impair opsonophagocytic killing, so vaccine-induced immunity reduces carriage acquisition and transmission less effectively (Choi et al., 2016; Luck et al., 2020). This is compounded by the expansion of specific, well-adapted lineages, as seen in our dataset where serotype 3 was concentrated within GPSC12, consistent with persistence driven by successful clonal backgrounds (Silva-Costa et al., 2021). The frequent detection of serotypes 8, 11A, 23B, 35B, and 9N further reflects established post-PCV serotype replacement patterns observed in other settings in both adult and paediatric populations (Richter et al., 2014; Lo et al., 2019; González-Díaz et al., 2020; Silva-Costa et al., 2021). Although country-specific differences in serotype composition and richness were observed, the substantial overlap in serotypes between Kuwait and the UAE suggests a shared circulating pneumococcal pool rather than distinct national populations.

However, marked age- and disease-associated differences in serotype distribution were observed. Serotypes 12F and 9V were significantly associated with invasive disease, in keeping with previous studies that identified serotype 12F as highly invasive and frequently implicated in post-PCV increases in invasive pneumococcal disease (Rokney et al., 2018; Ludwig et al., 2020). In contrast, serotype 11A and non-encapsulated pneumococci were significantly enriched among non-invasive isolates. Non-encapsulated pneumococci are well-recognized contributors to pneumococcal carriage and mucosal disease and are infrequently associated with invasive disease, consistent with their distribution in this study (Keller Lance et al., 2016). Among children, particularly those under 2 years of age, serotype diversity was more restricted, dominated by non-PCV13 serotypes such as 35B, suggesting ongoing serotype replacement in this age group. A comparative analysis across vaccine formulations demonstrated incremental gains in theoretical serotype coverage with increasing valency. While PCV13 and PCV15 covered a limited proportion of circulating isolates, particularly in children, PCV20 and PCV21 captured progressively larger proportions, with PCV21 providing comparable coverage across adult and paediatric populations. These coverage patterns have implications for regional vaccine policy. The limited coverage of contemporary isolates by PCV13 and PCV15, particularly in children under two years of age, suggests that current PCV formulations no longer match the circulating serotype pool. As PCV20 and PCV21 offer substantially broader coverage, the findings support a transition to higher-valency formulations for immunization programs in our setting. Additionally, genomic surveillance will be needed to track replacement and inform future adjustments in vaccine policy.

Assignment of isolates to GPSCs showed that the pneumococcal populations were dominated by globally distributed lineages rather than regionally restricted clones. The most prevalent GPSCs detected in both countries (including GPSC1, GPSC3, GPSC5, GPSC6, GPSC10 and GPSC59) correspond to well-characterized international lineages that have been widely reported across Europe, North America, and East and Southeast Asia (Gladstone et al., 2019). The presence of these globally disseminated GPSCs indicates that these pneumococcal populations are consistent with global transmission and extensive population movement, rather than country- or region-specific evolutionary patterns. This lineage-level analysis also provided important biological context, showing that although the pneumococcal population is distributed across 61 GPSCs, a small number of dominant lineages contribute disproportionately to MDR, invasive disease, and non-vaccine serotypes. GPSC10, which was mainly associated with serogroup 15 (15B/C) and consisted entirely of MDR isolates, was predominant in both countries. This finding reflects the global emergence of serogroup 15 lineages in the post-PCV era (Mendes et al., 2014; Richter et al., 2014; Lo et al., 2019; Chaguza et al., 2020). GPSC12 was associated with serotype 3 and contributed substantially to its persistence, consistent with evidence that serotype 3 success is driven by specific, well-adapted lineages (Silva-Costa et al., 2021, Silva-Costa et al., 2024). Other prevalent GPSCs, including GPSC6 and GPSC3, encompassed non-PCV13 serotypes and displayed heterogeneous resistance and disease associations, illustrating how lineage structure underpins serotype replacement dynamics. All major GPSCs included isolates from both countries, with no evidence of strict phylogeographic structuring. This observation was reinforced by pan-genome analysis, which showed identical total gene repertoires and comparable cloud gene content across isolates from both countries, indicative of a largely shared gene pool. Such patterns are consistent with previous genomic studies showing extensive cross-border dissemination of pneumococcal lineages and limited geographic compartmentalization (Gladstone et al., 2019; Lo et al., 2019). These patterns align with those reported elsewhere in the post-PCV era. The dominant lineages identified here are among the most prevalent GPSCs in the Global Pneumococcal Sequencing project’s worldwide analyses (Gladstone et al., 2019) rather than being regionally restricted. Similarly, the serotypes driving replacement in our setting mirror the non-vaccine serotypes expanding in other geographical areas (Weinberger et al., 2011; Balsells et al., 2017). This concordance with geographically distant populations reinforces the interpretation that regional pneumococcal epidemiology is shaped by global lineage circulation. A systematically matched genomic comparison across regions was beyond the scope of this study and is an important direction for future work.

An additional dimension of pneumococcal population dynamics observed was evidence of capsular switching within established sequence types, highlighting the ongoing role of homologous recombination in pneumococcal adaptation. Recombination-mediated replacement of the *cps* locus is a well-established mechanism by which *S. pneumoniae* can adapt in vaccine-pressured settings while retaining otherwise successful genomic backgrounds (Croucher et al., 2011; Golubchik et al., 2012; Wyres et al., 2013; Chaguza et al., 2020). Five STs were each identified in association with two distinct serotypes, consistent with putative capsule-switching events. These serotype pairs included both vaccine and non-vaccine types, illustrating how serotype variation can arise within otherwise stable genetic backgrounds through exchange at the *cps* locus. Among these events, robust genomic evidence for recombination-mediated capsule switching was demonstrated for GPSC5 (ST172), involving a switch from serotype 19A to serotype 35B. Our findings suggest a recent capsule-switching event rather than gradual lineage divergence, consistent with previously reported literature (Croucher et al., 2011; Golubchik et al., 2012; Wyres et al., 2013; Croucher et al., 2015; Chaguza et al., 2020). The direction of switching observed within GPSC5 is epidemiologically relevant in the context of post-PCV serotype replacement: serotype 19A is included in PCV13 and higher-valency formulations, whereas serotype 35B is a non-PCV13 serotype that has been increasingly reported in the post-vaccine era, particularly among paediatric populations (Richter et al., 2014; Desai et al., 2015). The temporal separation of these ST172 isolates, collected two years apart, is suggestive of, but does not date, the occurrence of capsule switching. The remaining serotype–ST pairings lacked definitive *cps*-spanning recombination signals and were therefore conservatively classified as putative. Nevertheless, the presence of multiple STs associated with more than one serotype, including within lineages contributing to MDR and non-vaccine serotype circulation, is consistent with prior genomic studies demonstrating that capsule switching is a recurrent feature of pneumococcal evolution in vaccine-pressured settings (Croucher et al., 2011; Wyres et al., 2013; Croucher et al., 2015). Importantly, the coexistence of vaccine and non-vaccine serotypes within the same lineage underscores the limitations of serotype-based surveillance alone and highlights the added value of integrating WGS to accurately interpret vaccine impact and anticipate future shifts in pneumococcal epidemiology.

Genomic evidence of MDR was a prominent feature of the pneumococcal population and was strongly structured at the lineage level. Resistance determinants were most frequently identified for β-lactams, macrolides, and tetracyclines, whereas those conferring resistance to fluoroquinolones and carbapenems remained rare, in keeping with previous reports (Richter et al., 2014; Kim et al., 2016). Several globally disseminated pneumococcal lineages, including GPSC1, GPSC10, GPSC6, GPSC9, and GPSC699, were uniformly MDR, indicating that the resistance burden in this setting is largely driven by the expansion of a limited number of high-risk clones rather than by widespread independent acquisition of resistance determinants. Similar lineage-associated resistance patterns have been described in large-scale pneumococcal genomic studies (Gladstone et al., 2019; Lo et al., 2019). These findings underscore the importance of lineage-resolved genomic surveillance for monitoring pneumococcal AMR and informing antimicrobial stewardship strategies. As the resistance burden was concentrated within a small number of globally disseminated MDR lineages (GPSC1, GPSC10, GPSC6, GPSC9 and GPSC699) rather than distributed evenly, local susceptibility data will remain crucial for guiding empirical therapy for suspected pneumococcal infection. In addition, lineage-resolved genomic surveillance could serve as an early-warning tool, with local expansion of these high-risk GPSCs signaling the need to reassess empirical guidance and vaccine-impact estimates.

Although this study represents key genomic analysis of *S. pneumoniae* from this under-represented region, a number of limitations should be acknowledged. The retrospective design relied on clinical isolates rather than population-based sampling, potentially introducing selection bias, and asymptomatic carriage was not assessed. Future studies should incorporate larger, population-based collections that include carriage isolates and linked clinical outcome data to improve the resolution of uncommon serotypes and provide a more comprehensive understanding of pneumococcal epidemiology in the region. In conclusion, pneumococcal populations circulating in the United Arab Emirates and Kuwait are embedded within global post-conjugate vaccine dynamics, characterized by serotype replacement, persistence of serotype 3, and dominance of internationally disseminated lineages with lineage-associated AMR profiles. The shared pan-genome structure, overlapping GPSC composition, and absence of country-specific genomic signatures suggest that transmission in this region is driven primarily by global connectivity, with disease burden being sustained by expansion of non-vaccine serotypes within established clonal backgrounds and recombination-mediated capsule diversification. These findings demonstrate that serotype distribution alone is insufficient to define pneumococcal epidemiology in highly connected settings. Lineage-resolved genomic surveillance must become integral to vaccine policy evaluation and AMR monitoring, particularly in global mobility hubs where local dynamics can both influence and be influenced by international transmission networks.

## Data Availability

The datasets presented in this study can be found in online repositories. The names of the repository/repositories and accession number(s) can be found in the article/**Supplementary Material**. Raw sequencing data generated in this study have been deposited in the NCBI Sequence Read Archive (SRA) under BioProject accession number PRJNA1417587.
