# Supplementary material for: Genomic epidemiology of Streptococcus pneumoniae reveals globally disseminated lineages and capsular switching in the Arabian Gulf region
Source: Front Cell Infect Microbiol. 2026 Sep 3;16:1832677. doi: 10.3389/fcimb.2026.1832677 (PMC13581929; doi:10.3389/fcimb.2026.1832677)
Supplement: Supplementary file 1 [file Table1.docx]

**Supplementary Table 1: Summary of Serotype-specific associations with invasiveness and age**

| **Serotype (N)** |  | **Invasive (Inv) vs_Non-Invasive (Non_Inv)** | | | | |  | **Adult_vs_Paediatric** | | | | |  | | **Paediatric** | | | | |
| --- | --- | --- | --- | --- | --- | --- | --- | --- | --- | --- | --- | --- | --- | --- | --- | --- | --- | --- | --- |
|  | **Inv N=103** | | **Non-Inv N=147 (%)** | **Odds ratio** | **95% CI** | **P value** | **Adult N=155 (%)** | | **PediatricN=95 (%)** | **Odds ratio** | **95% CI** | **P value** | | **<2 yrs N=43** | | **>2 yrs N=207** | **Odds ratio** | **95% CI** | **P value** |
| 3 (33) | 17 (16.5%) | | 16 (10.9%) | 1.62 | 0.78–3.37 | 0.25 | 28 (18.1%) | | 5 (5.3%) | 3.97 | 1.48–10.67 | 0.003* | | 1 (2.3%) | | 32 (15.5%) | 0.13 | 0.02–0.98 | 0.02* |
| 9V (4) | 4 (3.9%) | | 0 (0.0%) | 13.34 | 0.71–250.57 | 0.027* | 4 (2.6%) | | 0 (0.0%) | 5.67 | 0.30–106.57 | 0.3 | | 0 (0.0%) | | 4 (1.9%) | 0.52 | 0.03–9.83 | 1 |
| 11A (15) | 1 (1.0%) | | 14 (9.5%) | 0.09 | 0.01–0.72 | 0.005* | 10 (6.5%) | | 5 (5.3%) | 1.24 | 0.41–3.75 | 0.79 | | 2 (4.7%) | | 13 (6.3%) | 0.73 | 0.16–3.35 | 1 |
| 12F (5) | 5 (4.9%) | | 0 (0.0%) | 16.47 | 0.90–301.27 | 0.01* | 5 (3.2%) | | 0 (0.0%) | 6.98 | 0.38–127.68 | 0.16 | | 0 (0.0%) | | 5 (2.4%) | 0.42 | 0.02–7.80 | 0.59 |
| 16F (9) | 3 (2.9%) | | 6 (4.1%) | 0.7 | 0.17–2.89 | 0.74 | 0 (0.0%) | | 9 (9.5%) | 0.03 | 0.00–0.51 | 0.0001* | | 3 (7.0%) | | 6 (2.9%) | 2.51 | 0.60–10.47 | 0.19 |
| 24B/24C/24F (7) | 3 (2.9%) | | 4 (2.7%) | 1.07 | 0.23–4.90 | 1 | 7 (4.5%) | | 0 (0.0%) | 9.65 | 0.54–170.87 | 0.046* | | 0 (0.0%) | | 7 (3.4%) | 0.31 | 0.02–5.48 | 0.61 |
| 35B (11) | 3 (2.9%) | | 8 (5.4%) | 0.52 | 0.13–2.01 | 0.53 | 5 (3.2%) | | 6 (6.3%) | 0.49 | 0.15–1.67 | 0.34 | | 5 (11.6%) | | 6 (2.9%) | 4.41 | 1.28–15.18 | 0.02* |
| NCC2_aliC_aliD (8) | 0 (0.0%) | | 8 (5.4%) | 0.08 | 0.00–1.39 | 0.02* | 2 (1.3%) | | 6 (6.3%) | 0.19 | 0.04–0.98 | 0.06 | | 3 (7.0%) | | 5 (2.4%) | 3.03 | 0.70–13.19 | 0.14 |

*statistically significant

**Supplementary Table 2. Country-stratified pan-genome composition of pneumococcal isolates**

| **Country** | **Number**  **of isolates** | **Total genes** | **Core genes**  **(≥95%)** | **Shell genes**  **(15–95%)** | **Cloud genes**  **(<15%)** |
| --- | --- | --- | --- | --- | --- |
| UAE | 153 | 4,316 | 1,418 | 875 | 2,023 |
| Kuwait | 97 | 4,316 | 1,550 | 702 | 2,064 |

*Core genes were defined as those present in ≥95% of isolates within each country-specific dataset; shell genes were present in 15–95% of isolates; cloud genes were present in <15% of isolates.*

**Supplementary Table 3: Coverage of pneumococcal conjugate vaccine (PCV) serotypes by age group**

| **Vaccine formulation** | **Overall n/N (%)** | **Adult ≥18 y**  **n/N (%)** | **Paediatric <18 y**  **n/N (%)** | **Paediatric <2 y**  **n/N (%)** | **p value† (Adult vs Paediatric)** | **p value† (Adult vs <2 y)** |
| --- | --- | --- | --- | --- | --- | --- |
| **PCV13** | 73/250 (29·2) | 55/155 (35·5) | 18/95 (18·9) | 5/43 (11·6) | 0·006 | 0·002 |
|  |  |  |  |  |  |  |
| **PCV15** | 82/250 (32·8) | 60/155 (38·7) | 22/95 (23·2) | 7/43 (16·3) | 0·013 | 0·006 |
|  |  |  |  |  |  |  |
| **PCV20** | 125/250 (50·0) | 90/155 (58·1) | 35/95 (36·8) | 13/43 (30·2) | 0·002 | 0·002 |
|  |  |  |  |  |  |  |
| **PCV21** | 163/250 (65·2) | 103/155 (66·5) | 60/95 (63·2) | 25/43 (58·1) | 0·682 | 0·368 |

† p values calculated using Fisher’s exact test.

**Supplementary Table 4. Coverage of PCV20 and PCV21 serotypes by age group and clinical presentation**

| **Group** | **PCV20 serotypes n/N (%)** | **PCV21 serotypes n/N (%)** | **p value† (PCV20 vs PCV21)** |
| --- | --- | --- | --- |
| Overall | 125/250 (50·0) | 163/250 (65·2) | <0·001 |
| Adult invasive | 49/78 (62·8) | 54/78 (69·2) | 0·499 |
| Adult non-invasive | 41/77 (53·2) | 49/77 (63·6) | 0·252 |
| Paediatric invasive | 11/25 (44·0) | 18/25 (72·0) | 0·085 |
| Paediatric non-invasive | 24/70 (34·3) | 42/70 (60·0) | 0·004 |
| Paediatric <2 years invasive | 1/3 (33·3) | 0/3 (0·0) | 1 |
| Paediatric <2 years non-invasive | 12/40 (30·0) | 25/40 (62·5) | 0·007 |

*p-values represent within-stratum comparisons between PCV20 and PCV21 coverage using Fisher’s exact test*.

**Supplementary Table 5: Recombination-supported and Putative capsule-switching events**

| **GPSC** | **ST(s) involved** | **Serotypes observed** | **Number of isolates** | **cps-overlapping recombination (Gubbins red tracks)** | **Visual support in Phandango** | **Interpretation** |
| --- | --- | --- | --- | --- | --- | --- |
| GPSC5 | ST172 | 19A, 35B | 13 | Multiple clear cps-spanning tracts | Yes (strong) | Recombination-supported capsule switching |
| GPSC1 | ST320 | 19A, 19F | 8 | Limited/ambiguous | Weak | Putative |
| GPSC699 | ST6359 | 9N, 15B | 12 | Single, short tract | Weak–moderate | Putative |
| GPSC10 | ST13727 | 15B, 15C | 27 | None detected | No | Putative (likely mutation-driven) |
| GPSC9 | ST63 | 15A, 23B | 8 | None detected | No | Putative |
